# Inguinal Bladder and Ureter Hernia *Permagna*: Definition of a Rare Clinical Entity and Case Report

**DOI:** 10.1155/2018/9705728

**Published:** 2018-09-30

**Authors:** Michela De Angelis, Guido Mantovani, Francesco Di Lecce, Luigi Boccia

**Affiliations:** ^1^Division of General and Hepatobiliary Surgery, Department of Surgical Sciences, Dentistry, Gynaecology and Paediatrics, University of Verona, Verona, Italy; ^2^Department of General Surgery, Carlo Poma Hospital, Mantua, Italy

## Abstract

**Background:**

Inguinoscrotal herniation of the bladder is a rare clinical entity, with a frequency between 0.5% and 4% of all inguinal hernias. The bladder can partially or entirely herniate into the inguinal canal; when the whole bladder and ureters migrate into the scrotum, it may cause urinary disorders.

**Case Presentation:**

A 62-year-old male patient presented with urinary disorders and right-sided inguinoscrotal hernia. Under clinical suspicion of bladder involvement in the inguinal canal, abdominal and pelvic computed tomography (CT) scan with endovenous contrast was performed, revealing a right inguinoscrotal hernia, containing the whole urinary bladder and the right pelvic ureter. Without violating the urinary bladder wall integrity, the content of the hernial sac was reduced into the abdominal cavity. Hernioplasty was performed by means of Lichtenstein's method.

**Conclusions:**

Ureteral involvement should be suspected when a clinical inguinal hernia is diagnosed concurrently with unexplained hydronephrosis, renal failure, or urinary tract infection, as in the case described. When suspected, the preoperative diagnosis, particularly with CT scan, is essential to avoid complications and to reduce risk of bladder and ureter injuries during hernia repair.

## 1. Introduction

Inguinal hernia repair is a regularly done surgical procedure, which may sometimes surprise the surgeon with its peculiar contents.

The urinary bladder can be contained in inguinoscrotal hernia and, more rarely, in perineal [1] or obturator [2] hernia. In 1951, Levin proposed the term of “scrotal cystocele” to indicate a large scrotal hernia of the urinary bladder [3].

Inguinoscrotal herniation of the bladder is a rare clinical entity, with a frequency between 0.5% and 4% of all inguinal hernias [4, 5]. It is more frequent among males, over 50 years old. The most important risk factors of scrotal bladder hernia include [6, 7] body mass index (BMI) > 30, past injuries of the pelvis, previous abdominal wall or pelvic surgery, bladder obstruction (prostatic hypertrophy, urethral stricture, etc.), and bladder wall diseases (chronic inflammation, malignancy) [8]. We report on a rare case of whole bladder and pelvic ureter herniation in a right inguinoscrotal hernia, preoperatively investigated with computed tomography (CT) scan and treated by prosthetic hernia repair.

The bladder can partially or entirely herniate into the inguinal canal; when the whole bladder and ureters migrate into the scrotum, it may cause obstruction, calculi, vesicoureteral reflux, hydronephrosis, infection, and acute renal failure [9, 10].

The aim of this case report was to present a rare anatomical variant, which the surgeon might encounter during inguinal hernia surgical repair. Inguinal bladder hernia must be suspected in high risk patients, in order to perform preoperative investigation to better plan surgical operation.

## 2. Case Presentation

A 62-year-old male patient was followed by the Department of Medicine, Division of Nephrology of Mantua Hospital for treatment of recurrent pyelonephritis. The patient was on oral anticoagulant treatment (Warfarin) for pulmonary embolism, stage I obesity was diagnosed with the BMI amounting to 32.4, and he also had right-sided inguinoscrotal hernia *permagna* since 4 years, never treated for patient decision.

The patient complained of symptoms during the last three weeks, such as dysuria, incomplete urinary bladder emptying, reduction of the size of the hernia after voiding, and increased urination after compression of the scrotal area.

The blood test shown haemoglobin 11.3 g/dl, hematocrit 34.9%, platelet count 354 × 10^9^/l, leukocytosis (13.5 × 10^9^/l), and increased creatinine level (1.52 mg/dl), with urea level 57 mg/dl, international normalized ratio (INR) of 2.39. Urinalysis demonstrated hematuria, 61 white cell count/hpf, and the presence of *Escherichia coli* in urine culture.

Nephrologist required surgical evaluation of that huge hernia. During the physical examination, the patient complained of pain and right-sided inguinal and scrotal discomfort with associated swelling in the ipsilateral testicle and scrotum. His abdomen was soft, nontender, and nondistended.

**Figure 1:**
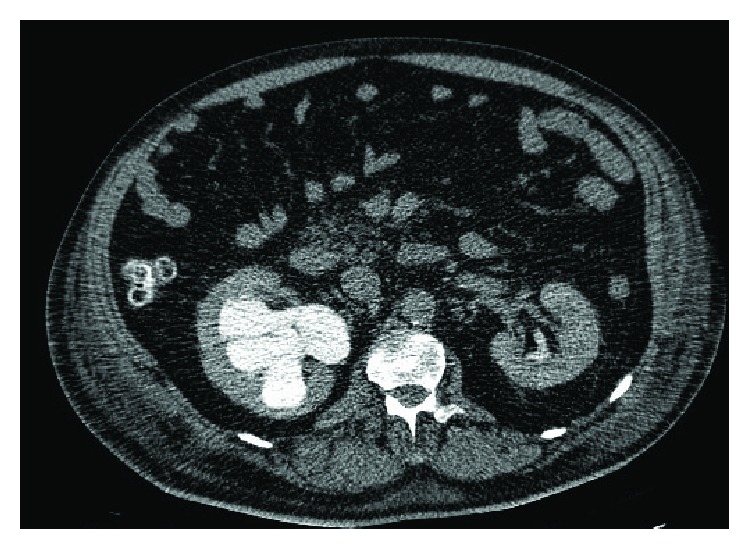
Abdominal-pelvic CT scan with endovenous contrast: right hydronephrosis with ipsilateral renal atrophy.

**Figure 2:**
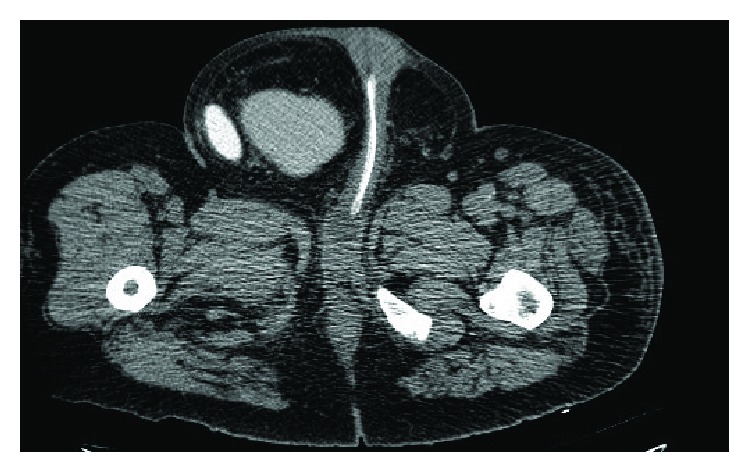
Abdominal-pelvic CT scan with endovenous contrast: right inguinal hernia, containing the whole urinary bladder and the right pelvic ureter dilated.

**Figure 3:**
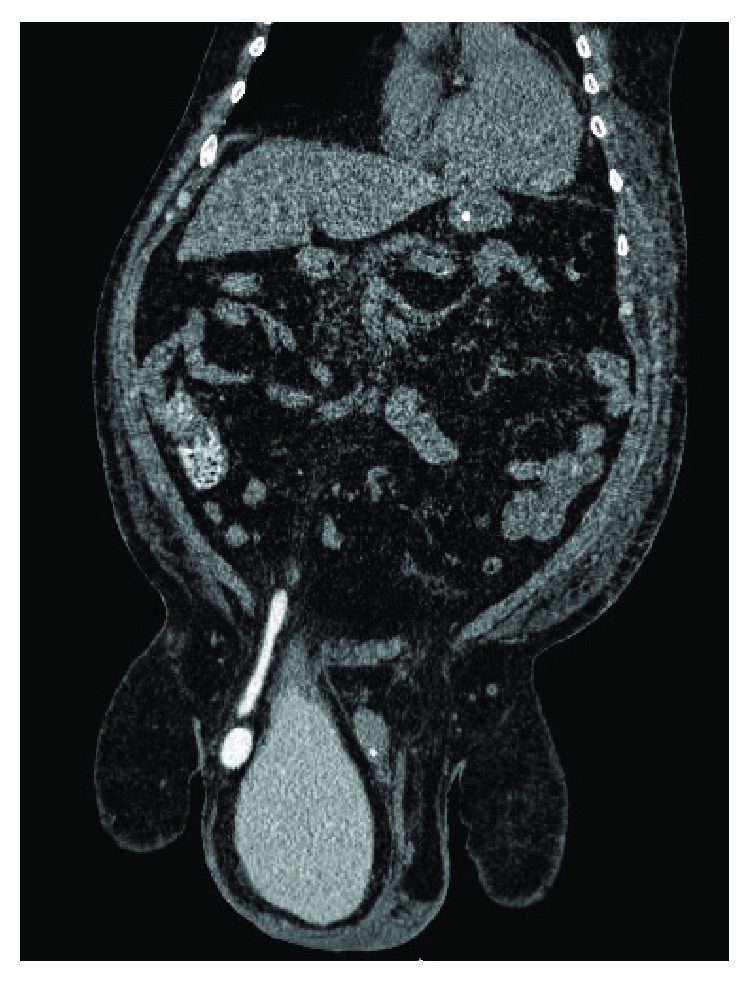
Abdominal-pelvic CT scan with endovenous contrast: right inguinal hernia, containing the whole urinary bladder and the right pelvic ureter dilated; coronal reconstruction.

**Figure 4:**
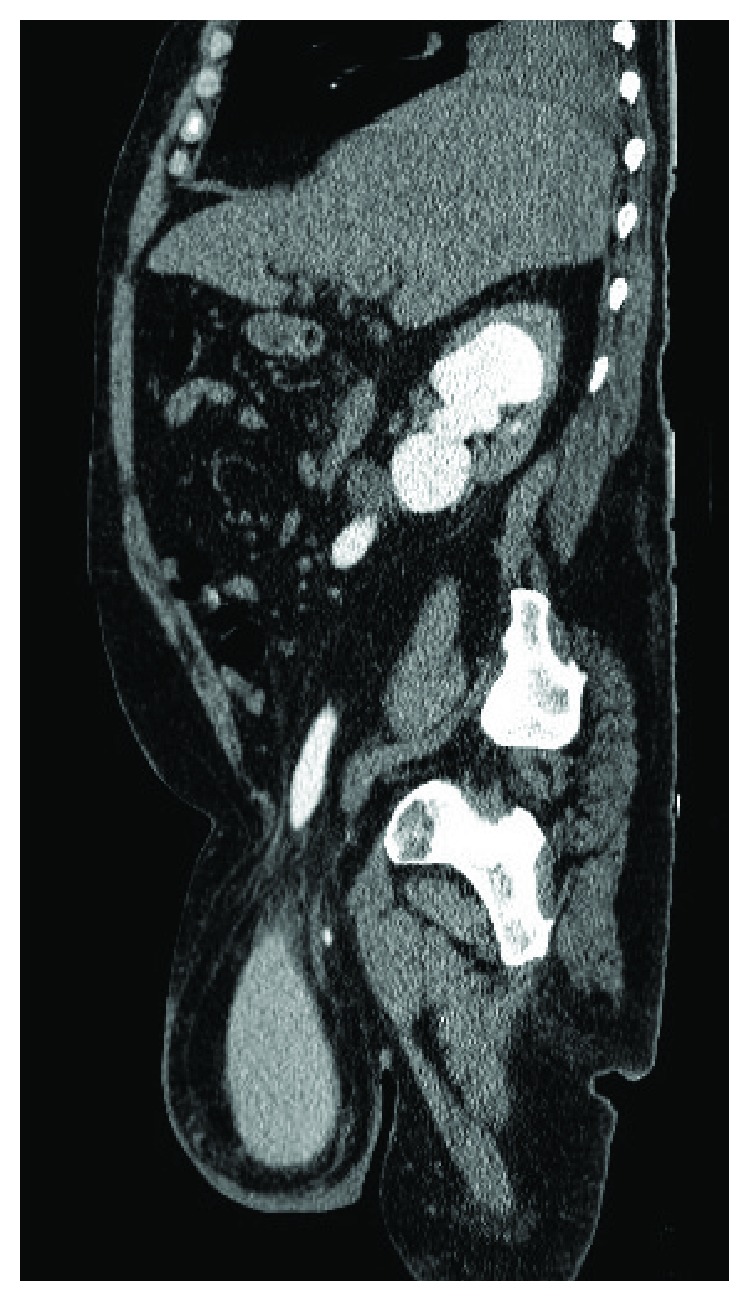
Abdominal-pelvic CT scan with endovenous contrast: right inguinal hernia, containing the whole urinary bladder and the right pelvic ureter dilated; sagittal reconstruction.

Under clinical suspicion of bladder inguinoscrotal hernia, a scrotal ultrasound and an abdominal and pelvic CT scan with endovenous contrast were performed, revealing a right inguinoscrotal hernia, containing the whole urinary bladder and the right pelvic ureter, associated with right hydronephrosis with ipsilateral renal atrophy (Figures 1–4).

Therefore, the patient was admitted to the Department of Surgery of Mantua Hospital for treatment of right-sided inguinoscrotal hernia. Before surgical intervention, Warfarin therapy was replaced with therapeutic low molecular weight heparin (LMWH), and antibiotic prophylaxis was performed.

A urethral catheter (18 Fr) was placed to decompress the bladder and to look for hematuria in the postoperative period.

**Figure 5:**
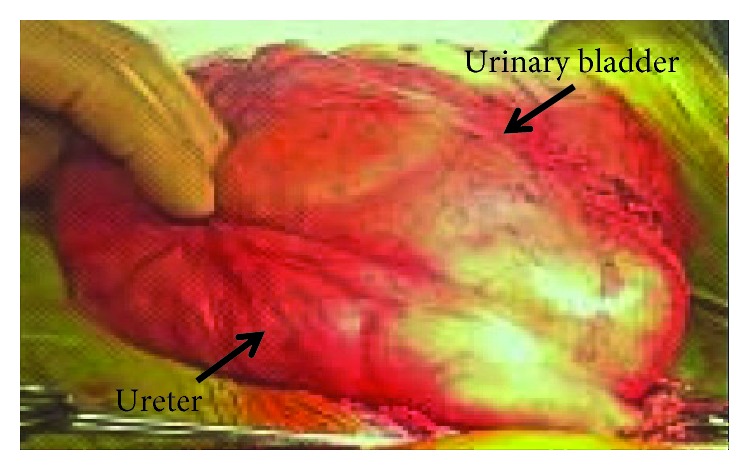
Intraoperative image of the bladder and the pelvic ureter filled with physiological solution, showing the dilatation of the right ureter (more than 3 cm in diameter).

After spinal anaesthesia, a right inguinal incision was performed. After opening the right inguinal canal, we detected a direct inguinal hernia with complete breakthrough of the inguinal canal posterior wall. We opened the hernial sac containing the bladder and right ureter. After a long and careful isolation of the elements of the spermatic cord, physiological solution (500 ml) was injected by the urethral catheter to fill the bladder and, consequently, the pelvic ureter, in order to better define hernia content. The right ureter was very dilated, with a maximum diameter of more than 3 cm, as shown in Figure 5, because of grade 4 vesicoureteral reflux.

The bladder and the right ureter were gently separated from the spermatic cord. Without violating the urinary bladder wall integrity, the content of the hernial sac was reduced into the abdominal cavity. Hernioplasty was performed applying a polypropylene mesh, 10 × 15 cm in size, by means of Lichtenstein's method. The postoperative period was uneventful. Five days after surgery, the patient was discharged from the hospital in good general condition. Patient follow-up, with clinical examination and ultrasound, at one month and one year did not reveal hernia recurrence neither urinary disorders.

## 3. Discussion

The hernia in the inguinal region usually contains the omentum and small intestine but rarely can contain unusual contents like the appendix [11], Meckel's diverticulum [12], ovary with fallopian tubes [13], sigmoid colon [14], and urinary bladder.

The bladder may herniate as an indirect or a direct inguinal hernia, respectively, through the internal deep inguinal ring or medially to the epigastric vessels. Among inguinal bladder herniation, the direct hernias are the most frequent.

Inguinoscrotal herniation of the bladder is a rare clinical entity, particularly when associated with herniation of the ureter. The condition is often diagnosed incidentally because most patients are asymptomatic or complain of nonspecific symptoms such as daytime frequency, scrotal mass, dysuria, and urgency [15, 16].

The most typical finding is two-stage voiding [17]: urine in the abdominal part of the bladder empties first, followed by the urine in the herniated part that usually needs manual compression. In most cases, the bladder herniation is diagnosed during surgical repair of inguinal hernias [2], because surgeons generally proceed to inguinal hernia surgery without imaging. Sometimes, it is diagnosed during the postoperative period, based mainly on the presence of complications such as persistent urinary bladder leakage and pathological urine secretion from the operative wound [18].

Preoperative physical examination, surgical alertness, and imaging diagnostics (US and CT) enabled to diagnose preoperatively scrotal cystocele [19–21].

Although the importance of preoperative diagnosis, less than 7% of bladder hernia are diagnosed preoperatively, while 16% are diagnosed postoperatively due to complications and the others are diagnosed intraoperatively [22]. A preoperative diagnosis of scrotal cystocele allows to place a urethral catheter, with the aim to decompress the bladder before surgery and to fill the bladder with saline fluid and a blue dye (methylene blue) intraoperatively, in order to help surgeon to better identify the hernial sac content and to preserve urinary structure integrity. Delayed diagnosis is often the cause of costly postoperative complications [23]. The risk of bladder injury was reported as 12% during the hernia repairs [24] and it leads to hematuria, sepsis, urinary leakage and fistula formation [7].

The presence of urinary tract cancer associated with scrotal cystocele is an anecdotal finding with few cases described in literature. A surgical review shown that 13 cases out of 116 patients with bladder herniation (11.2%) had a malignancy; 9 had bladder carcinoma and 4 prostate carcinoma [5]. Recently, other Authors confirmed this finding [25, 26]. Since this association, although rare, has been reported in the literature, we believe that when a bladder or ureter inguinal hernias are suspected, a CT scan has to be performed, while ultrasound does not have enough sensitivity and specificity.

## 4. Conclusions

Scrotal cystocele is very rarely encountered, but a high index of suspicion should be maintained for high-risk population, such as patients with inguinal hernia, particularly males, over 50 year old and obese, who have symptoms that indicate urological pathologies or with typical two-stage voiding. Ureteral involvement should be suspected when inguinal hernia is diagnosed concurrently with unexplained hydronephrosis, renal failure or urinary tract infection, as in the case described. When bladder and/or ureter inguinal hernias are suspected, CT scan has to be performed, because it allows identifying hernial sac content and eventually underlying urinary tract malignancies. Preoperative diagnosis is essential to guide surgical approach, reducing risk of bladder and ureter injuries during hernia repair and to avoid postoperative complications.
